# Quantitative Detection of Trace Malachite Green in Aquiculture Water Samples by Extractive Electrospray Ionization Mass Spectrometry

**DOI:** 10.3390/ijerph13080814

**Published:** 2016-08-11

**Authors:** Xiaowei Fang, Shuiping Yang, Konstantin Chingin, Liang Zhu, Xinglei Zhang, Zhiquan Zhou, Zhanfeng Zhao

**Affiliations:** 1 Jiangxi Key Laboratory for Mass Spectrometry and Instrumentation, East China Institute of Technology, Nanchang 330013, China; fxw273@126.com (X.F.); shpyang@ecit.cn (S.Y.); chingin.k@gmail.com (K.C.); liang.zhu1981@gmail.com (L.Z.); leizi8586@126.com (X.Z.); 2 Department of Electronic and Information Engineering, Harbin Institute of Technology, Weihai 264209, China; zzq@hitwh.edu.cn

**Keywords:** extractive electrospray ionization, rapid detection, malachite green, water, mass spectrometry

## Abstract

Exposure to malachite green (MG) may pose great health risks to humans; thus, it is of prime importance to develop fast and robust methods to quantitatively screen the presence of malachite green in water. Herein the application of extractive electrospray ionization mass spectrometry (EESI-MS) has been extended to the trace detection of MG within lake water and aquiculture water, due to the intensive use of MG as a biocide in fisheries. This method has the advantage of obviating offline liquid-liquid extraction or tedious matrix separation prior to the measurement of malachite green in native aqueous medium. The experimental results indicate that the extrapolated detection limit for MG was ~3.8 μg·L^−1^ (S/N = 3) in lake water samples and ~0.5 μg·L^−1^ in ultrapure water under optimized experimental conditions. The signal intensity of MG showed good linearity over the concentration range of 10–1000 μg·L^−1^. Measurement of practical water samples fortified with MG at 0.01, 0.1 and 1.0 mg·L^−1^ gave a good validation of the established calibration curve. The average recoveries and relative standard deviation (RSD) of malachite green in lake water and *Carassius carassius* fish farm effluent water were 115% (6.64% RSD), 85.4% (9.17% RSD) and 96.0% (7.44% RSD), respectively. Overall, the established EESI-MS/MS method has been demonstrated suitable for sensitive and rapid (<2 min per sample) quantitative detection of malachite green in various aqueous media, indicating its potential for online real-time monitoring of real life samples.

## 1. Introduction

Malachite green (MG) is a cationic triarylmethane dye that is commonly used as a biocide in aquaculture worldwide. It provides efficient defense against fungal attacks, protozoan infections and other diseases in aquatic organisms, e.g., caused by helminths [1]. Besides that, MG is extensively used as a food coloring agent, medical disinfectant, and industrial dye (e.g., in silk, wool, paper, etc.) [1,2]. However, MG and its metabolite, leucomalachite green (LMG), can remain in aquatic animal tissues and the aquiculture environment for a long time, which is of concern since it has been reported to cause carcinogenesis, mutagenesis, chromosomal fractures, teratogenicity and respiratory toxicity [1]. In China, the limit of detection of MG in aquiculture animal tissue is 2 μg·kg^−1^ using an official method (national standard GB/T 19857-2005 of PR China). In the EU, the use of MG for food fish was banned in 2000 [1,3]. Inspection of illegal MG usage also requires high-throughput measurements of surface and ground water samples, especially when the presence of MG residuals was confirmed in aquaculture products in that area. In addition, due to the bioaccumulation in fish and other aquatic animals, MG residues in tissue of aquaculture products are much more prominent compared to the determined MG level in fish farm effluents [4], posing more challenges on analytical methods. In Ireland, the concentration of MG in fish farm water should be below 100 μg·L^−1^ [5]. Hence, rapid detection of MG in water is of great importance from both the perspectives of human health and environmental preservation.

Over the years, an increasing number of analytical techniques such as spectrophotometry [6,7,8], high performance liquid chromatography (HPLC) [9,10,11], capillary electrophoresis Raman spectroscopy (CE-RS) [12], liquid chromatography tandem mass spectrometry (LC/MS^n^) [2,13,14] and RNA-Aptamer–based assay [15] have been adapted for the detection of MG in various water matrices. Although the aforementioned techniques are considered as routine MG detection techniques, they are time-consuming and require complicated sample pretreatment (e.g., extraction, pre-concentration, derivatization, etc.). For example, low concentrations of MG and LMG in water samples have been detected using maghemite nanoparticles as the pre-concentration material, followed by spectrophotometric detection [6]. The limit of detection (LOD) was found to be 0.28 μg·L^−1^ after the complicated pre-concentration processes (maghemite synthesis, adsorption and desorption processes), which took more than 2.5 min per sample [6]. Temperature-controlled ionic liquid dispersive liquid–liquid microextraction combined with high performance liquid chromatography was also introduced to analyze MG in environmental water, with a LOD as low as 0.086 μg·L^−1^ [9]. A long extraction time (~50 min) is necessary to achieve such a performance. In this regard, a rapid, reliable and sensitive technique for MG identification in environmental samples would be more beneficial.

Liquid samples can be directly analyzed by extractive electrospray ionization mass spectrometry (EESI-MS) without sample pretreatment [16,17,18,19,20]; thus, it has been gradually extended to analysis of samples in various physical states, such as solid, gas and aerosol [21]. In this study, the EESI-MS/MS method for the rapid detection of MG has been developed using a homemade EESI source combined with an ion-trap multistage mass spectrometer. Rapid quantitative detection of MG in aqueous matrices has been demonstrated with high speed, simplicity and a good recovery rate.

## 2. Materials and Methods

### 2.1. Materials and Reagents

Malachite green was purchased from Tianjin Fuchen Chemical Reagent Factory. Methanol (HPLC grade) was provided by ROE Company (Newark, DE, USA). Ultrapure water (resistivity 18.2 MΩ·cm) was supplied by a Barnstead Nanopure ultrapure water purification system (ThermoFisher Scientific, Boston, MA, USA). Environmental water samples were obtained from a man-made lake (pH 5.5) and aquariums for feeding *Carassius carassius* (pH 6.0), respectively.

### 2.2. EESI-MS Condition

**Figure 1:**
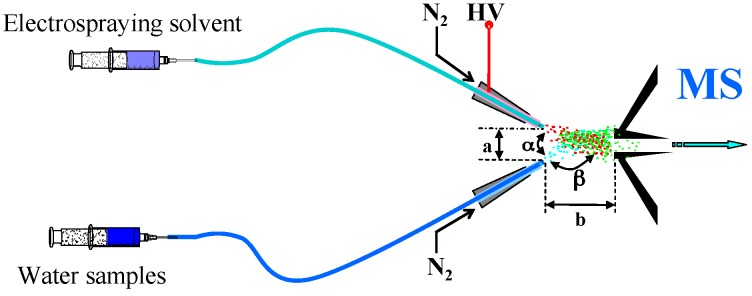
Schematic diagram of the EESI source.

Experiments were carried out using a LTQ-XL mass spectrometer (Finnigan, San Jose, CA, USA) equipped with a home-made EESI source [21,22,23]. The EESI source and the LTQ mass spectrometer were set to work in positive-ion detection mode. MS spectra were recorded in the range of 50–500 *m/z.* The ESI voltage was set at 3.5 kV; the temperature of the ion-transport capillary was 400 °C; the injection rates of ESI solvent and sample solution was set at 3 μL·min^−1^ and 5 μL·min^−1^, respectively; high purity nitrogen gas (purity ≥ 99.999%) from a gas cylinder are used for nebulizing the ESI solvent (methanol; silica capillary, i.d. 0.1 mm) and sample solution (silica capillary, i.d. 0.1 mm); and the pressure was 1.4 MPa. The EESI assembly was mounted on a 3-D adjustable stage (shown in Figure 1). The distance (a) between the two channels of the EESI source and the distance (b) between the tips of the EESI source and the MS inlet were optimized to be 1 mm and 5 mm, respectively. The angle (α) between the two sprays and the angle (β) between individual sprays and the MS inlet were around 60° and 150°, respectively. 

The full scan mass spectra were recorded using Xcalibur software of the LTQ-MS instrument. In collision induced dissociation (CID) experiments, the ion at *m*/*z* 329 was selected as the parent ion, and the isolation width and activation time were set at 1.5 Da and 30 ms, respectively. CID was set with 30% collision energy, and other parameters were automatically optimized by LTQ-MS system. All the mass spectra were recorded with an average duration time of 0.2 min, followed by background subtraction.

### 2.3. Preparation of Spiked Samples

Stock solution of malachite green (1 g·L^−1^) was prepared by dissolving 1 g of malachite green in the 1 L volumetric flask of ultrapure water, and stored in dark. Standard working solutions (1–10,000 μg·L^−1^) were prepared by serially diluting the malachite green stock solution with lake water. 

## 3. Results and Discussion

### 3.1. Detection of Malachite Green by EESI-MS

**Figure 2:**
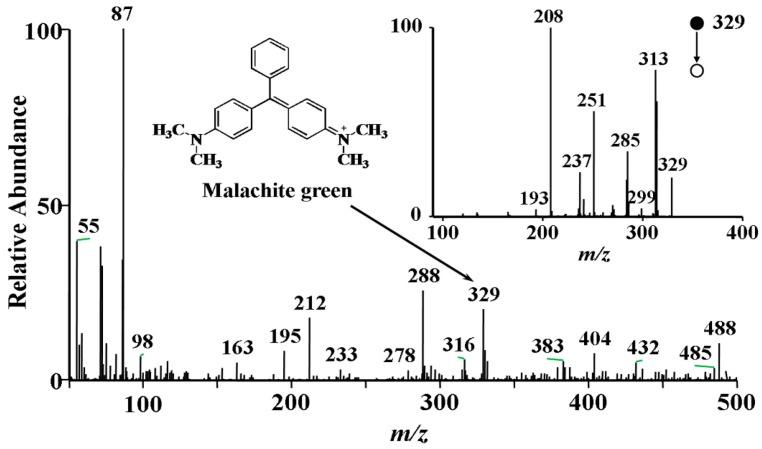
EESI-mass spectra of 0.1 mg·L^−1^ malachite green obtained directly from water sample. The inset shows the MS/MS spectrum of malachite green (*m*/*z* 329).

EESI ionization relies on the microscopic liquid-liquid extraction between the spray of neutral analyte droplets and the spray of primary ions. Produced secondary ions, subsequent to the solvent dissolvation process, are then directly sampled to the inlet of a mass spectrometer for mass interrogation. Figure 2 shows the EESI-MS spectrum of a pure water sample spiked with 0.1 mg·L^−1^ MG. In the MS^2^ spectrum of the MG cation (*m*/*z* 329), major fragments at *m*/*z* 313, 285, 251, 237, 208 were recorded upon collision activation at the energy of 30% (the inset of Figure 2), which is in a good agreement with previous observations [24]. These characteristic fragments are likely to be produced via the neutral losses of CH_4_, C_2_H_6_N, C_6_H_6_, C_7_H_8_ and C_8_H_11_N, respectively.

### 3.2. Optimization of EESI Parameters

In order to achieve the best extraction and ionization efficiency of malachite green within aqueous samples using our EESI source, several experimental parameters, including ESI voltage, sample injection rate, ion-transport capillary temperature and sheath gas (N_2_) pressure, were systematically optimized.

#### 3.2.1. Electrospray Voltage

**Figure 3:**
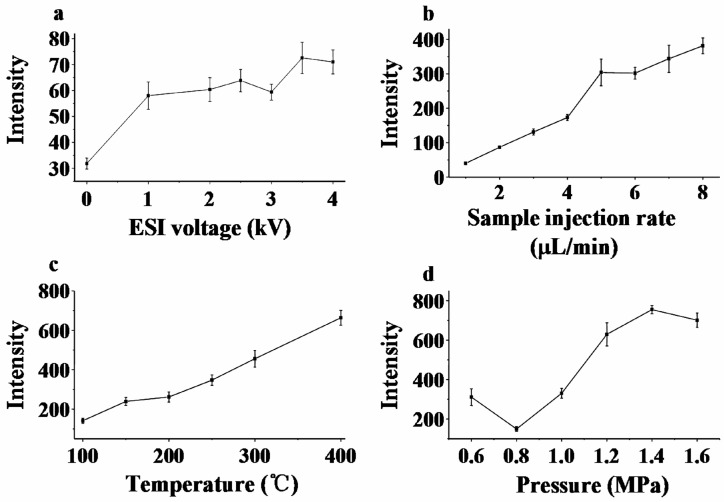
Variation of the signal intensity with the ESI voltage (**a**); sample injection rate (**b**); ion-transport capillary temperature (**c**); and nebulizing gas (N_2_) pressure (**d**).

The impact of the ionizing electrospray voltage on the signal intensity of the characteristic fragment *m/z* 208 is shown in Figure 3a. The higher the voltage, the stronger the MG signal that was observed. However, corona discharge occurred between the tips of the two ESI channels when the electrospray voltage was beyond 3.5 kV. Thus, in this study, the ESI voltage of 3.5 kV was used for the MG analysis to get the most stable and intense signal.

#### 3.2.2. Sample Flow Rate

The signal intensity of characteristic fragment *m*/*z* 208 was found to grow with the sample infusion rate (Figure 3b). Because a very high flow rate of the sample injection can cause contamination of the instrument, we used the value of 5 μL·min^−1^ in this work which avoids the contamination of the MS and gets the desired sensitivity.

#### 3.2.3. Temperature of the Heated Capillary

The desolvation process of charged droplets can be facilitated by the elevated temperature of the ion-transport capillary, resulting in a better efficiency of producing gaseous species [25]. Accordingly, the signal intensity of the *m*/*z* 208 signal increased with the temperature (Figure 3c). No heat-induced fragmentation was observed for MG ions at capillary temperatures up to 450 °C.

#### 3.2.4. Sheath Gas Pressure

Based on previous experience, the crucial factor which determines the nebulization effect is the ratio of the gas-liquid volume at the end of the spray. Therefore, controlling the sheath gas pressure to optimize the signal intensity of characteristic fragment *m*/*z* 208 is important and key, as shown in Figure 3d. The higher the pressure, the better the efficiency of the sample nebulization, which is particularly important for aqueous samples. In our experiments we chose the optimum sheath gas pressure as 1.4 MPa (room temperature 20 °C, velocity 568 m/s). At higher pressures, serious disturbance of the online liquid-liquid extraction/ionization plume was observed due to the extremely high gas flow velocity. 

### 3.3. Quantification of Malachite Green in Lake Water

**Figure 4:**
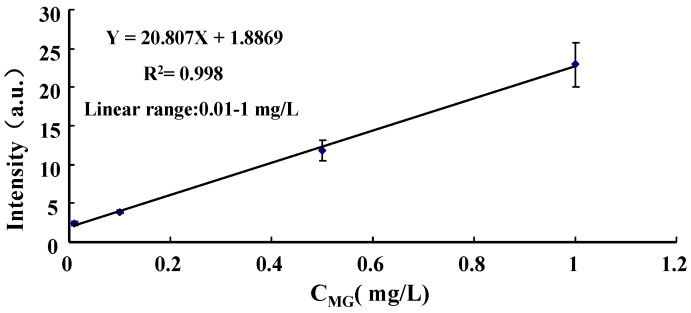
Dependence of the signal intensity on MG concentration in lake water.

Under the optimized experimental parameters, lake water samples spiked with 0.001, 0.01, 0.1, 0.5, 1, 5 and 10 mg·L^−1^ of MG were analyzed by EESI-MS and EESI-MS/MS and blank pure water samples were run as the background signal. Each standard solution was replicated six times independently. The MG concentration dependence for the average signal intensity of MS/MS fragment *m*/*z* 208 was plotted with the background subtracted. The mean values of six measurements with standard deviation (SD) and relative standard deviation (RSD) as error bars were 0.50 (0.029, 5.8%), 2.4 (0.30, 13%), 3.9 (0.18, 4.5%), 12 (1.3, 11%), 23 (2.8, 12%), 1.9 × 10^2^ (11, 6.0%) and 1.1 × 10^3^ (68, 5.9%), respectively, for lake water samples. As shown in Figure 4, the equation y = 20.807x + 1.8869 with a R^2^ = 0.998 at 95% confidence limits was obtained for MG in the range of 0.01–1.0 mg·L^−1^. Since the points correspond to the concentrations of 5 and 10 mg·L^−1^ beyond the linear range, the two points have been excluded in the fitting process. The LOD for the lake water sample was extrapolated as 3.8 μg·L^−1^ (S/N = 3), while the value for the spiked ultrapure water batch was estimated to be ~0.5 μg·L^−1^ (S/N = 3) (data not shown). While MG can be determined in water samples by LC-vis/FLD and LC-MS/MS with lower LODs of 50 ng·L^−1^ and 40 ng·L^−1^, respectively [2], the higher detection sensitivity in those analyses is achieved at the cost of time-consuming sample pretreatment steps. The LOD of EESI-MS/MS can be greatly improved if potentially combined with organic extraction (such as solid-phase extraction (SPE), liquid-liquid micro-extraction (LLME)) and subsequent pre-concentration. As demonstrated, the proposed EESI-MS/MS technique here can be taken as an efficient and reliable technique for the purpose of both monitoring illegal MG usage in aquaculture and water quality management in aquatic ecosystems, taking the analysis speed and ease of operation offered into account. Further research aiming at improving the sensitivity of the current EESI-MS/MS method is in progress; thus, it will pave the way for the direct determination of MG residues in various aquaculture media.

### 3.4. Real-Life Sample Analyses

Two different water samples, lake and aquarium water used to feed *Carassius carassius*, were quantitatively analyzed using the developed method. Although the background in the mass spectra of the lake water is neater than that of the fish water due to the more complex inclusions of the latter, MG was not detected in any of these water samples, indicating that the levels of MG in these water samples were below the detection limit. In order to calculate the recovery, spiked samples were prepared at the MG concentrations of 0.1 mg·L^−1^, 0.01 mg·L^−1^ and 1.0 mg·L^−1^. Due to the matrix effect and system error, the recovery rates deviated 100% and were close to 100%, which shows the selectivity and roughness of our method. In addition, due to the same reason mentioned above, the recovery rate of the fish water deviated 100% further. These results are listed in Table 1.

### 3.5. Sample Consumption and Analysis Speed

Low sample consumption is of great value for the analysis of real-life samples that are difficult to obtain. In this study, the minimum volume of the sampled solution was below 1 mL. The measurement time in a typical experiment was less than 2 min. Due to the minimal requirements for the sample’s pretreatment, the measurement time has been greatly reduced in comparison with LC-MS. This shows that the EESI-MS/MS method has several advantages such as high accuracy, high sensitivity and low sample consumption for rapidly quantifying trace analytes in a complex matrix.

## 4. Conclusions

In this work, a novel method based on EESI-MS/MS has been developed and applied to detect the trace levels of malachite green in different types of aquiculture waters without sample pre-treatment. The analysis does not require tedious sample preparation and can be accomplished within less than 2 min per sample. The method can potentially be applied for the detection of MG and its metabolites from a variety of complex matrices (water, urea, blood, fish tissues and animal feed, etc.) with high throughput.

## Figures and Tables

**Table 1:** Analytical results of spiked samples (*n* = 6).

Sample	Amounts Added (mg·L^−1^)	Amounts Measured (mg·L^−1^)	Relative Standard Deviation (RSD, %)	Recovery (%)
Lake water	0.100	0.115	6.64	115
Fish water ^a^	0.0100	0.00854	9.17	85.4
Lake water	1.00	0.960	7.44	96.0

^a^ the water from an aquarium for feeding *Carassius carassiu.*
